# Population-Based Stroke Atlas for Outcome Prediction: Method and Preliminary Results for Ischemic Stroke from CT

**DOI:** 10.1371/journal.pone.0102048

**Published:** 2014-08-14

**Authors:** Wieslaw L. Nowinski, Varsha Gupta, Guoyu Qian, Wojciech Ambrosius, Radoslaw Kazmierski

**Affiliations:** 1 Biomedical Imaging Lab, Singapore Bioimaging Consortium, Agency for Science Technology and Research, Singapore, Singapore; 2 Department of Neurology, Poznan University of Medical Sciences, Poznan, Poland; 3 Department of Neurology and Cerebrovascular Disorders (L. Bierkowski Hospital), Poznan University of Medical Sciences, Poznan, Poland; INSERM U894, Centre de Psychiatrie et Neurosciences, Hopital Sainte-Anne and Université Paris 5, France

## Abstract

**Background and Purpose:**

Knowledge of outcome prediction is important in stroke management. We propose a lesion size and location-driven method for stroke outcome prediction using a Population-based Stroke Atlas (PSA) linking neurological parameters with neuroimaging in population. The PSA aggregates data from previously treated patients and applies them to currently treated patients. The PSA parameter distribution in the infarct region of a treated patient enables prediction. We introduce a method for PSA calculation, quantify its performance, and use it to illustrate ischemic stroke outcome prediction of modified Rankin Scale (mRS) and Barthel Index (BI).

**Methods:**

The preliminary PSA was constructed from 128 ischemic stroke cases calculated for 8 variants (various data aggregation schemes) and 3 case selection variables (infarct volume, NIHSS at admission, and NIHSS at day 7), each in 4 ranges. Outcome prediction for 9 parameters (mRS at 7th, and mRS and BI at 30th, 90th, 180th, 360th day) was studied using a leave-one-out approach, requiring 589,824 PSA maps to be analyzed.

**Results:**

Outcomes predicted for different PSA variants are statistically equivalent, so the simplest and most efficient variant aiming at parameter averaging is employed. This variant allows the PSA to be pre-calculated before prediction. The PSA constrained by infarct volume and NIHSS reduces the average prediction error (absolute difference between the predicted and actual values) by a fraction of 0.796; the use of 3 patient-specific variables further lowers it by 0.538. The PSA-based prediction error for mild and severe outcomes (mRS = [2]–[5]) is (0.5–0.7). Prediction takes about 8 seconds.

**Conclusions:**

PSA-based prediction of individual and group mRS and BI scores over time is feasible, fast and simple, but its clinical usefulness requires further studies. The case selection operation improves PSA predictability. A multiplicity of PSAs can be computed independently for different datasets at various centers and easily merged, which enables building powerful PSAs over the community.

## Introduction

Knowledge of outcome prediction is important in effective stroke management [1]. The ability to estimate prognosis is important in stroke treatment decisions, particularly, with the advent of novel therapies, such as intra-arterial thrombolysis [2] and new stent retrievers [3], [4]. Outcome prediction can help in the planning of discharge, rehabilitation, end-of-life care, and patient and/or family communication and counselling [5]. Numerous approaches have been proposed for stroke outcome prediction [5]–[28]. They are based on a statistically significant correlation among patient-specific parameters, such that the patient-specific outcomes are predicted based on some independent variables measured for the same patient, mostly without accounting for infarct location. Despite the availability of numerous prognostic models, risk scores and prediction rules, none has gained widespread use in clinical practice [6].

The existing stroke prediction methods can be classified as a “same-patient-different-parameters” type or model. Here we propose a conceptually different model, namely, “same-parameter-different-patients”. Furthermore, we introduce a novel stroke outcome prediction method based on the “same-parameters-different-patients” model. This method is lesion size and location-driven and uses a Population-based Stroke Atlas (PSA). The rationale for PSA-based prediction is to use the aggregated information from similar cases (patients) to predict an outcome for a new case. The PSA is a means of aggregating data and knowledge from the previously treated patients with a preferable long follow up (to enable long-term predictions) and applying them to the currently treated patients. The PSA links neurological examination parameters with pathology localized on diagnostic neuroimages for a population of stroke patients. It aggregates a multiplicity of parameters and presents the distribution of each parameter as a three-dimensional (3D) image volume. These 3D volumes can be processed, analyzed and visualized as well as knowledge, trends and predictions extracted from them. Any PSA is a collection of population-based stroke maps (PSMs), each map calculated for a single parameter to be predicted. The predicted parameter distribution is obtained by getting it from the PSA in the normalized infarct region of the predicted case.

The purpose of this work (which is an extended version of our preliminary work presented at the International Stroke Conference ISC 2012 [29]) is 1) to introduce a method for calculation of the PSA and 2) to study PSA properties for different data aggregation and data selection schemes. To study PSA-based prediction properties, we introduce (*i*) PSA variants to account for various spatial mutual configurations of ischemic infarct outlines (i.e., different data aggregation schemes) and (*ii*) constrained PSA to accommodate for selection of suitable or relevant cases forming the PSA (i.e., different data selection schemes). We additionally demonstrate examples of PSA use illustrated by preliminary results of outcome prediction in ischemic stroke measured in terms of modified Rankin Scale (mRS) and Barthel Index (BI).

## Materials and Methods

### Material

A cohort of generally treated stroke patients with a large number (for some patients up to 170) of neurological parameters per patient, noncontrast CT (NCCT) scan at admission, and one year follow up was acquired. The Hospital Bioethics Committee’s approval was obtained (The Ethics Committee of the Poznan University of Medical Sciences, Poznan, Poland – decision no. 167/07, dated 01 Feb 2007; the title of approval “Clinical, laboratory and neuroimaging predictive factors in stroke”) and all scans were anonymized. From this cohort, a group of 458 consecutive ischemic stroke patients were selected (the baseline characteristics of this group were described in detail earlier [10]). The neurological parameters included history, hospitalization, demographics, laboratory parameters, clinical measures and outcomes. The scans were acquired on Picker PQ2000/5000 scanners with KVP 120 kV, tube current 200 mA, and reconstruction slice 5 mm. Outcome measurements in terms of mRS and BI were assessed for up to one year after stroke onset. From this group, cases suitable to build a preliminary version of the PSA were selected. This selection was limited to the cases with clearly visible ischemic infarcts that could be delineated. Cases with a complete set of data and at least one year patient’s survival (and if not available, then the longest possible) were preferable. Moreover, the cases with a midline shift, leukoaraiosis and old infarcts as well as hemorrhages and edemas causing anatomical distortion were excluded. The strict process resulted in selecting for this study a dataset of 128 cases of neurologically confirmed ischemic strokes with all the infarcts delineated (contoured) earlier (as part of another study [30]). The numbers of cases for mRS and BI scores at different days are given in Table 1. The time from stroke onset along with the number of the corresponding cases were: below 3 hours (10 cases) between 3–8 hours (52 cases), above 8 hours (66 cases), and above 24 hours (33 cases). The mean ± standard deviation (SD) of NIHSSa (National Institutes of Health Stroke Scale (NIHSS) at admission) = 8.3±6.6, range = [0–31]. The mean ± SD of NIHSS7 (NIHSS at day 7) = 6.9±7.2, range = [0–30]. The NIHSS means of infarcts in the left/right hemispheres were (8.83/7.25) and those for NIHSS7 (7.11/6.12). The mean of infarct volumes for the left/right hemispheres were (25.96 cm^3^/20.45 cm^3^). The mean ± SD of patients’ age were 64.6±12.5. The mean ± SD of patients’ mRS(7;30;90;180;360) (i.e., mRS at 7th, 30th, 90th, 180th and 360th day) and BI(30;90;180;360) (i.e., BI at 30th, 90th, 180th and 360th day) were (2.9±1.7;2.4±2.1;2.04±2.1;2.02±2.2;2.2±2.3) and (73.0±33.7;83.8±25.1;86.8±21.5;87.0±21.2), respectively. The mean ± SD size of the NCCT scans (in MB) were 11.87±0.80, range = [10–15.5].

**Table 1:** Numbers of cases for mRS (upper part) and Barthel Index (lower part).

mRS at day	Number of cases with mRS = 6	Number of cases with mRS<6	Unavailable values
7	1	125	2
30	6	119	3
90	11	111	6
180	15	103	10
360	18	98	12
**Barthel Index at day**	**Number of cases with Barthel Index = 0**	**Number of cases with Barthel Index>0**	**Unavailable values**
30	7	112	9
90	1	110	17
180	2	101	25
360	2	97	29

### Method

**Figure 1:**
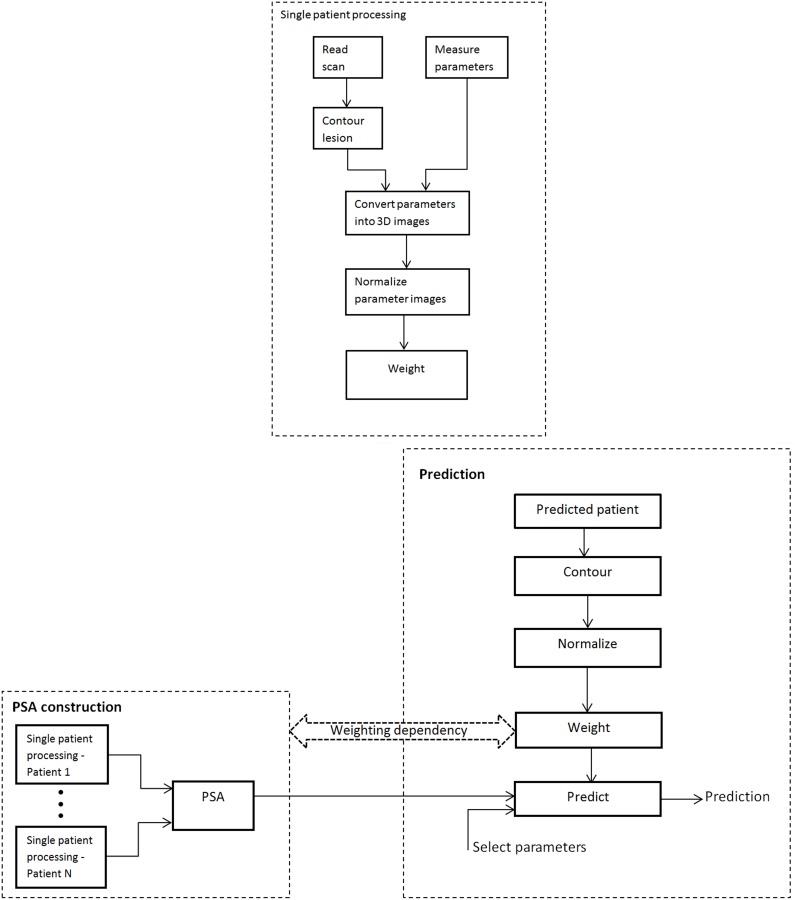
Illustration of PSA calculation and outcome prediction. Top) processing of a single patient (case) contributing to formation of the PSA. Bottom) formation of the PSA from its contributing patients (left) and PSA-based prediction (right). The horizontal arrow represents weighting dependency between the PSA and a predicted case.

The method has two stages: 1) calculation of the PSA, and 2) PSA-based patient-specific outcome prediction, as diagrammed in Figure 1. The PSA is built for a set of predictable parameters, where a parameter is a neurological parameter, scan density (intensity) or its characteristics, or generally any computable entity. A high level description of the algorithm for PSA calculation is the following.

*For each parameter*
*For each case/scan*
*Contour the infarct(s) (create the contour file)*
*Normalize spatially the contour file*
*For each voxel within the normalized contour file*
*Aggregate the parameter value*
*Divide the aggregated values by the number of the contributing cases*

The ischemic infarcts were contoured manually on all the scans by using a dedicated contour editor [31]. This tool provides means to create and edit contours in the acquisition (axial) plane; display coronal and sagittal planes; and window images, including the routine head (30,80) and acute stroke (30,30) windows in Hounsfield units. For each case, the complete set of contours (called a contour file) delineating the whole ischemic lesion region was generated.

Each case was spatially normalized by projecting it to a common stereotactic (Talairach) space of 512×512×64 voxels and 0.320119×0.320119×2 mm^3^ resolution [32]. We used a landmark-based, fast normalization method which employs the midsagittal plane extracted by using the algorithm by Volkau et al. [33] and the modified Talairach landmarks [34] calculated by employing a statistical approach detailed in [35].

For each studied parameter, its corresponding PSM was calculated by aggregating the parameter’s value within each contour file across all spatially normalized cases and by dividing the aggregated value in each voxel by the number of contributing cases (i.e., the number of contour files containing this voxel). The simplest way to aggregate data was to assign the parameter value to all the voxels within the contour file and accumulating them across all cases, which resulted in a spatial distribution of the average value of a considered parameter. In general, the process of parameter value aggregation shall take into account the size, overlap and distance of the contour files, both these which form the PSA and that under prediction.

An instant of PSM (i.e., a PSA for a given parameter) can be calculated for all the cases or any subset of them selected by determining the case selection variables. This selection operation determines the contour files used for the construction of the PSM by including relevant and/or excluding unsuitable cases. For this study, three simple, patient-specific case selection variables were applied and examined here: infarct volume, NIHSS at admission (NIHSSa) and at 7^th^ day (NIHSS7), although, generally, any other variables can be chosen for analysis.

To study the impact on outcome prediction of various relationships between the infarct regions (contours files) forming the PSA and the infarct region of the case under prediction, we created eight variants of PSA. They differ in the way how the contour files are combined when forming the PSA, meaning if they are amplified or dampened depending on their overlap, PSA contour file volume, contour file volume of the to-be-predicted case, and/or contour localization. The variants were calculated by applying weighting with eight weights denoted as *w*_1_,…,*w*_8_.

Let *PSM_p,k_* denote the population-based stroke map for parameter *p* calculated by applying the *k*-th weight to each normalized contour file *C_i_*, *i* = 1,…,*N*, where *N* is the number of cases forming this *PSM*. Then
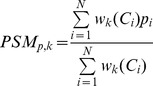
(1)


The weighs are defined as follows:
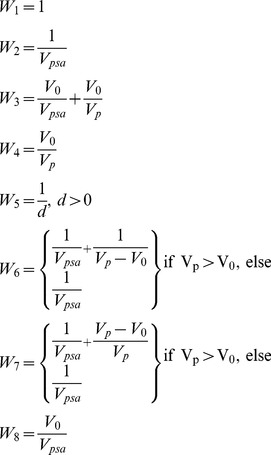
(2)where *Vpsa* is the volume of a PSA contour file, *Vp* is the volume of the infarct region of the case under prediction, *Vo* is the volume of the overlap of the PSA infarct region and that under prediction, and *d* is the distance between the centroids of the PSA infarct region and that under prediction.

The interpretation of the weights is as follows. Weight *w*_1_ produces averaging of the parameter value (meaning that no weighting is applied). Weight *w*_2_ causes more weightage to smaller (i.e., with a better localization) PSA contours (this weighting is also applied as a component in weights *w*_3_ and *w*_6_–*w*_8_). Weights *w*_3_–*w*_4_ and *w*_8_ give more weightage to the PSA contour files with a higher overlap or closer (weight *w*_5_) to the contour file of the to-be-predicted case; note that the knowledge of the latter is required prior to the PSA calculation (i.e., for *w*_3_–*w*_8_ weights). Weights *w*_6_–*w*_7_ take into account the difference between the infarct and overlapped regions.

As the rationale for PSA-based prediction is to use the aggregated information from similar cases to predict an outcome for a new case, a high level description of the algorithm for patient-specific outcome prediction is the following (see also Figure 1 bottom).

*Contour the infarct(s) of a case under prediction (create the contour file)*
*Normalize the contour file of the case under prediction*
*Weight contours*
*Obtain PSA parameter characteristics from within this normalized contour file*

### Analyses

Two main types of analyses were carried out: 1) to study the properties of the PSA in terms of data aggregation and data selection (2 analyses); and 2) to evaluate the prediction capabilities of the preliminary PSA in terms of individual mRS scores and dichotomical classification for mRS and BI scores (2 analyses).

We used a leave-one-out prediction approach to obtain preliminary prediction results. Then, each case (patient) was predicted based on the PSA constructed from the remaining 127 cases, meaning that a predicted case was not included into a construction of the corresponding PSA used for prediction. A single PSM instance was calculated for each of 8 weights, 3 ischemic infarct volume thresholds resulting in 4 volume ranges (whole range, ≤8.0 cm^3^ (at 50th volume percentile), ≤25.9 cm^3^ (at 75th volume percentile), and ≤70 cm^3^), and 4 selections for each NIHSSa and NIHSS7, each with two thresholds of 5 and 13 resulting in 4 ranges ([0–42], [0–5], [6]–[13] and [14]–[42]). These NIHSS ranges are associated with the following predictions: discharge home [0–5], rehabilitation [6]–[13], and nursing facility care [14]–[42] [22). Therefore, 512 PSM instances had to be calculated per case and per predicted outcome parameter. For a single case, 9 parameters were predicted: mRS7, mRS30, mRS90, mRS180, mRS360, BI30, BI90, BI180, and BI360. To predict all 128 cases, 589,824 PSMs were calculated. Each PSM required processing all but one scans. In other words, during these analyses the scans were processed 74,907,648 times.

The prediction accuracy error to be minimized was defined as the absolute difference of the actual outcome parameter value (known for this patient from the follow-up) and the predicted value (as calculated by our method) for the studied parameter of the considered patient. Effects of different PSA variants and case selection variables on the prediction error were examined to select the best variants and variables. Note that mRS and BI vary not only across time but also across value (mRS = [0–6], BI = [0–100]), which substantially increases the number of combinations to be analyzed. Moreover, the narrower the selection range, the lower the statistical power and potentially the higher the influence of outliers.

To assess the PSA variants (i.e., different ways of PSA construction in terms of data aggregation) on the outcome prediction error, we combined the mRS7, mRS30, mRS90, mRS180 and mRS360 parameters and calculated the prediction error. Student’s t-statistic and 2-tailed p-value assessed significant differences between the errors corresponding to the different variants.

The effect of case selection variables on error was evaluated in 4 situations; namely for: infarct volume; NIHSSa; infarct volume and NIHSSa; and infarct volume, NIHSSa and NIHSS7. The error reduction ratio, defined as the prediction with variable selection to that without variable selection, was calculated for 2 and 3 variables. The best variables, defined as the most frequent values in the first quartile (≤25th percentile) error range, were determined for individual prediction at mRS = 0,…,6 along with the resulting errors (note that the lowest error values were not used to avoid outliers).

To assess PSA performance in distinguishing favourable from unfavourable outcomes, we measured the area under the Receiver Operating Characteristic (ROC) curve. The mRS predictions calculated for all 3 variables were dichotomized as: 1) 0–2 (favourable outcome) and 3–6 (unfavourable outcome), and 2) 0–1 (excellent outcome) and 2–6 (unfavourable outcome) [36]. This analysis was repeated for BI dichotomized as [0–45] and [46–100] [37].

## Results

**Figure 2:**
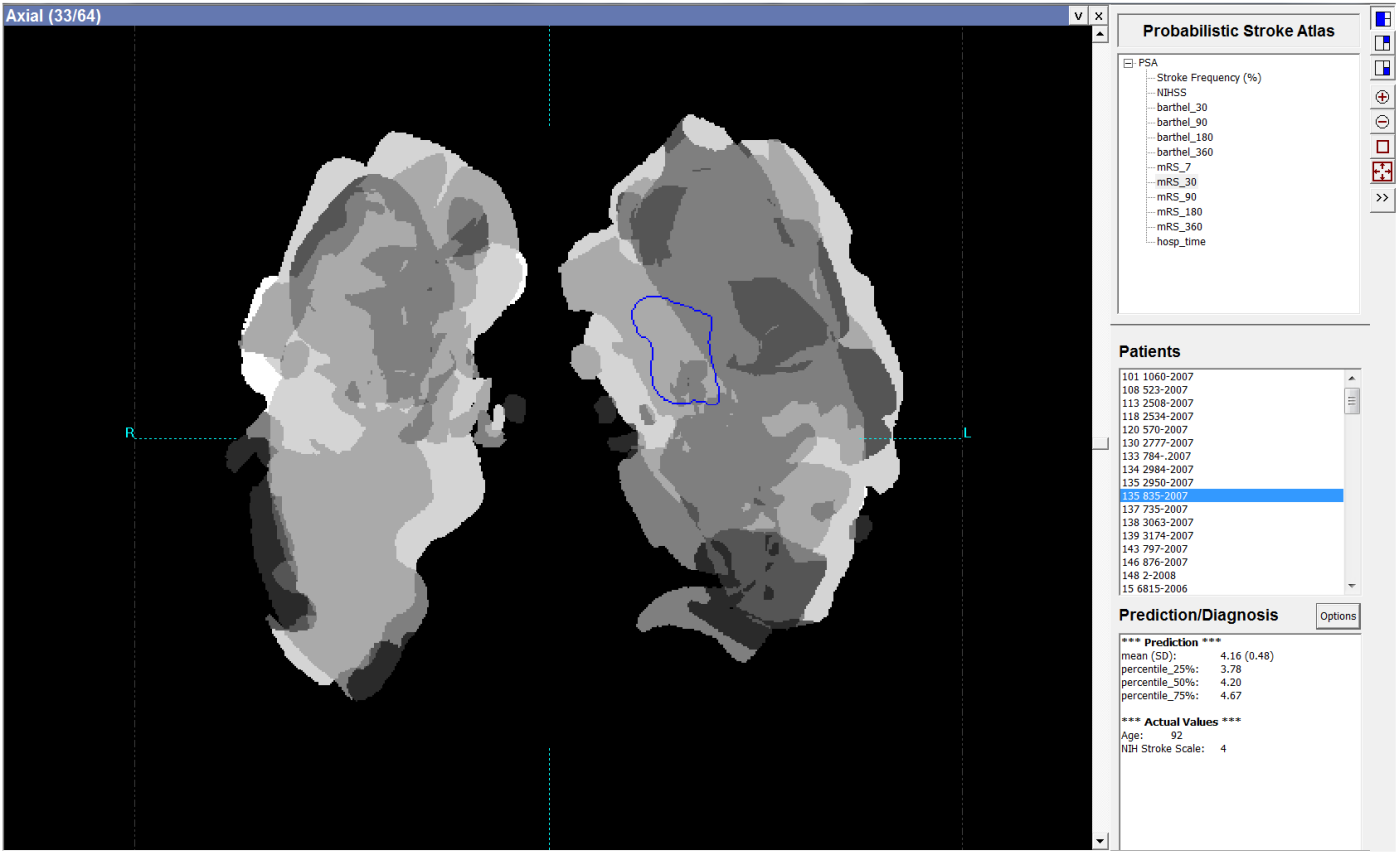
The software platform for PSA calculation and illustration of PSA-based prediction. The calculated maps of interest and the cases (patients) to be predicted are selectable from the first two top panels on the right. For illustration, the mRS90 map is selected here and shown as an axial image along with the superimposed normalized contour of the case under prediction (in the left hemisphere). The results of mRS90 prediction (the mean value of 4.25) along with the actual value for this patient (of 4) are shown in the right-bottom panel.

**Figure 3:**
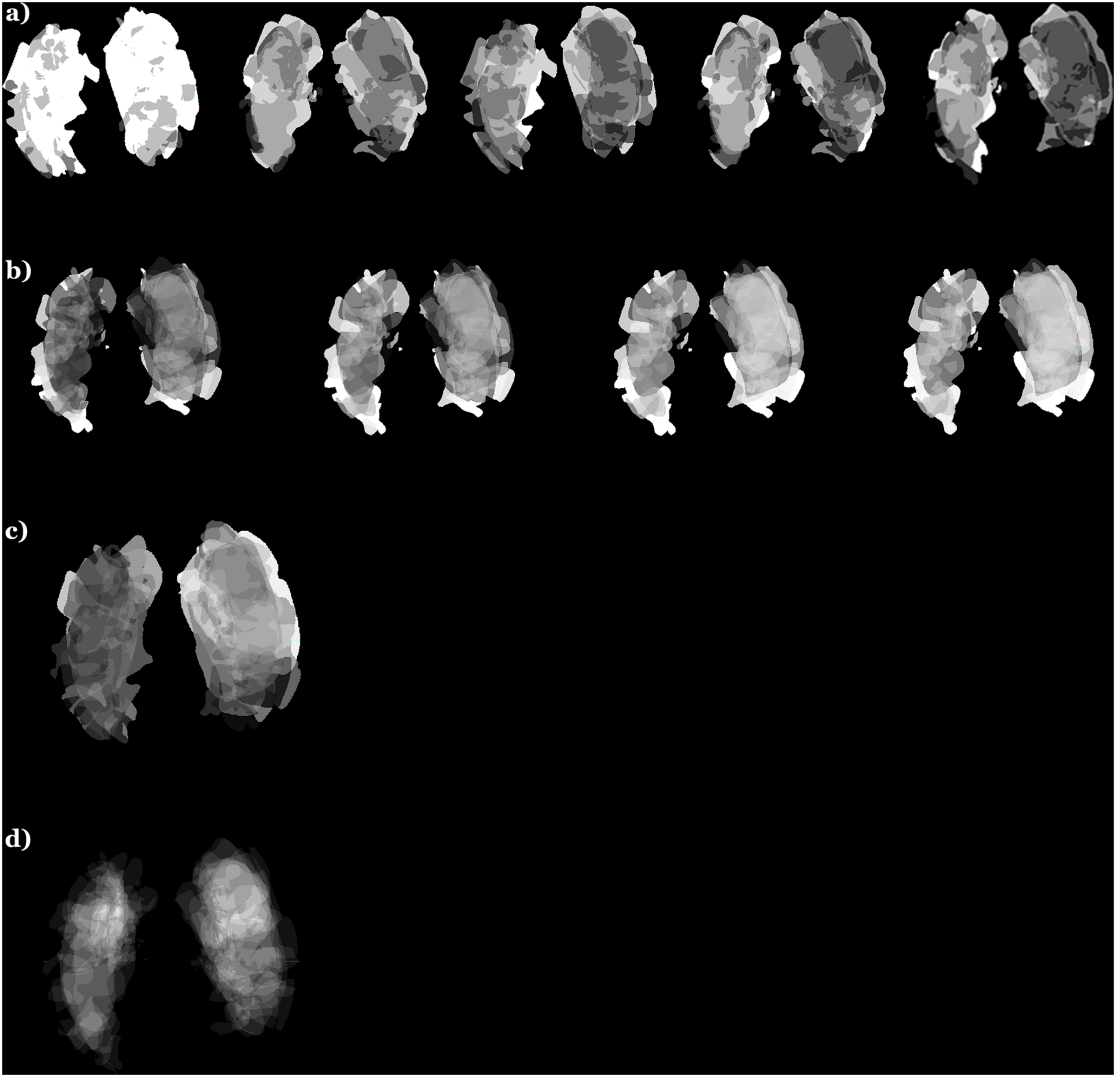
Examples of PSA maps calculated for *w_1_* weighting (i.e., parameter and scan averaging): a) mRS (from the left to the right mRS7, mRS30, mRS90, mRS180, mRS360); b) BI (from the left to the right BI30, BI90, BI180, BI360); c) NIHSS at admission (note that the left hemisphere intensity of the NIHSS map is higher than that of the right hemisphere corresponding to the fact that patients with a right sided ischemic stroke are associated with a lower NIHSS score [45]); d) NCCT image intensity (infarct frequency) distribution. Image intensity, proportional to map value, was normalized to 0–255 range. Note the trends over time in the similar locations of the mRS and BI maps (demonstrating the decrease in intensity for mRS and the increase in intensity for BI) which correspond to the improvement of outcomes over time (as the patients with up to one year survival were included). The images are in the radiological convention.

A software platform to calculate PSAs and provide PSA-based prediction was developed, and its user interface is shown in Figure 2. The results of analyses described in Section 2.3 are presented here. By using this software platform, the preliminary version of the PSA was calculated for the predicted parameters (including mRS7, mRS30, mRS90, mRS180, mRS360, BI30, BI90, BI180, BI360), and (for illustration) for NIHSS at admission and NCCT image density (infarct frequency) distribution, Figure 3.

The overall average mRS accuracy results are summarized in Table 2 providing the prediction errors and their standard deviations with respect to the PSA variants and case selection variables for the mRS scores combined across time (mRS7,…,mRS360) and value in two situations mRS = [0–6] and mRS = [2]–[5]. The two tailed p-value for pairwise variant comparison is >0.22 implying that the variants are statistically similar. Table 2 also gives the error reduction ratios for 2- and 3-variable selection and the average values across variants.

**Table 2:** Overall average mRS results (the prediction error and its standard deviation) with respect to variants (weights *w_1_,…,w_8_*) and case selection parameters (infarct volume, NIHSSa and NIHSS7) for the mRS scores combined across time (mRS7,…,mRS360) and value in two situations mRS = [0–6] (top row) and mRS = [2]–[5] (bottom row in brackets).

Variant(weight)	All selected	Volume selection	NIHSSa selection	Volume and NIHSSa selection	Volume, NIHSSa, and NIHSS7 selection	3-variable error reduction ratio	2-variable error reduction ratio
*W_1_*	1.933±1.238	1.549±1.029	1.854±1.246	1.132±0.759	0.933±0.561	0.483	0.586
	(1.081±0.663)	(0.772±0.326)	(1.039±1.039)	(0.597±0.117)	(0.524±0.045)	(0.485)	(0.552)
*W_2_*	1.831±1.006	1.698±0.927	1.642±0.904	1.259±0.790	0.940±0.575	0.513	0.688
	(1.103±0.452)	(1.041±0.425)	(0.967±0.313)	(0.724±0.187)	(0.571±0.571)	(0.518)	(0.656)
*W_3_*	1.969±1.261	1.549±0.978	1.905±1.222	1.570±0.874	0.919±0.418	0.467	0.797
	(1.144±0.684)	(0.823±0.297)	(1.091±0.643)	(0.867±0.308)	(0.612±0.053)	(0.535)	(0.758)
*W_4_*	1.967±1.330	1.514±1.010	1.898±1.287	1.558±0.895	0.884±0.395	0.450	0.792
	(1.145±0.767)	(0.788±0.341)	(1.085±0.719)	(0.853±0.345)	(0.623±0.074)	(0.544)	(0.745)
*W_5_*	1.853±1.140	1.541±0.946	1.721±1.118	1.517±0.867	0.927±0.500	0.500	0.819
	(1.055±0.537)	(0.825±0.259)	(0.957±0.521)	(0.823±0.272)	(0.628±0.054)	(0.595)	(0.780)
*W_6_*	1.829±0.993	1.702±0.916	1.803±0.989	1.862±0.877	1.492±0.842	0.816	1.018
	(1.109±0.437)	(1.052±0.414)	(1.088±0.436)	(1.057±0.365)	(0.637±0.071)	(0.574)	(0.953)
*W_7_*	1.812±1.022	1.688±0.943	1.625±0.965	1.594±0.853	0.806±0.410	0.445	0.880
	(1.067±0.440)	(1.003±0.402)	(0.909±0.35)	(0.706±0.190)	(0.619±0.063)	(0.580)	(0.662)
*W_8_*	1.792±1.009	1.618±0.919	1.668±0.985	1.404±0.748	1.120±0.664	0.625	0.784
	(1.044±0.411)	(0.925±0.296)	(0.971±0.412)	(0.708±0.109)	(0.678±0.052)	(0.649)	(0.678)
Average	**1.885±0.400**	**1.603±0.339**	**1.769±0.388**	**1.487±0.295**	**1.003±0.199**	**0.538**	**0.796**
	**(1.096±0.564)**	**(0.904±0.350)**	**(1.013±0.598)**	**(0.792±0.254)**	**(0.612±0.059)**	**(0.560)**	**(0.723)**

The 2- and 3-variable error reduction ratios due to case selection are given. Moreover, the average values across the weights are provided.

The best selection variables for mRS scores and the corresponding average errors are given in Table 3.

**Table 3:** Best selection variables for mRS scores and the corresponding mean errors and standard deviations (the maximum volume is 250.99 cm^3^).

mRS	Volume(cm^3^)	NIHSSa variable range	NIHSS7 variable range	Mean absolute error	Error standard deviation
0	≤8.008	0–5	0–5	2.273	1.043
1	≤8.008	0–5	0–5	1.275	0.882
2	≤8.008	0–5	0–5	0.690	0.572
3	≤70	14–42	6–13	0.699	0.614
4	≤max volume	14–42	0–42	0.524	0.462
5	≤max volume	14–42	14–42	0.624	0.561
6	≤max volume	14–42	14–42	1.894	0.707

The areas under ROC curves corresponding to a dichotomical favourable versus unfavourable classification for mRS and BI are given in Table 4.

**Table 4:** Favourable/excellent versus unfavourable outcome prediction for 3 variable case selection for the *w_1_* weight.

Time(in days)	Modified Rankin Scale		Barthel Index
	[0–2] vs [3]–[6]	[0–1] vs [2]–[6]	[0–45] vs [46–100]
7	0.779[0.688–0.854]	0.704[0.608–0.789]	-
30	0.710[0.608–0.798]	0.720[0.620–0.807]	0.748[0.650–0.830]
90	0.657[0.550–0.753]	0.663[0.557–0.758]	0.774[0.675–0.855]
180	0.668[0.560–0.765]	0.664[0.556–0.762]	0.850[0.755–0.919]
360	0.573[0.459–0.681]	0.582[0.468–0.690]	0.653[0.536–0.756]

The areas under ROC (95% confidence interval) for mRS and BI corresponding to different times.

PSA-based prediction took 8.44±1.13 seconds (s) computed on a Dell Precision WorkStation 390; OS: Microsoft Windows XP Professional SP3; CPU: Intel Core2 Quad Processor Q6600, 2.40 GHz, 4 GB RAM. Most of this time was spent for landmark detection (6.06±0.92 s).

## Discussion

The key objective of this work was to introduce a new class of a stroke outcome prediction method and to study its properties from two standpoints: data aggregation and data selection. In addition, we evaluated prediction capabilities of the preliminary PSA in terms of individual mRS and BI scores as well as dichotomical classification.

### PSA-based prediction

The proposed prediction method belongs to a class of “same-parameter-different-patients”, is infarct size and location-driven and combines neurological data with neuroradiology imaging. The analyses carried out here assumed that a predicted case was known prior to the PSA calculation, so the prediction and computation of the PSA were performed simultaneously. This assumption allowed us to study different ways of PSA creation (data aggregation) expressed in terms of variants (weights). Although intuitively the results of weighting should vary (as the weights depend on multiple factors, including the size of overlap (of the normalized ischemic lesions), PSA contour file volume, contour file volume of the to-be-predicted case, and/or distance between the contour centroids), the prediction outcomes of all PSA variants are statistically equivalent (at least for the data used in this study). This feature has several important consequences. First, the use of the simplest weight *w_1_* is feasible resulting in the fastest PFA calculation. Second, the PSA can be pre-calculated before prediction, which is not feasible when employing the *w*_3_–*w*_8_ weights. Third, a multiplicity of PSAs can be computed independently for different datasets (and possibly at various centers) and easily merged, which opens a possibility of building powerful PSAs over the community. Fourth, although this analysis covered all 8 weighting schemes and required excessive data, future studies of PSA can disregard weighting.

The average prediction error of mRS [2]–[5] is around one grade (1.096±0.564) and a 3-parameter selection lowers it to about half a grade (0.612±0.059). The PSA constrained by 2-variable case selection reduces the average prediction error by a fraction of 0.796 for mRS = [0–6] (or 0.723 for mRS = [2]–[5]), whereas 3-variable selection (feasible at day 7th) further lowers this error by a fraction of 0.538 (or 0.560), see Table 4. This result indicates that PSAs customized to certain situations or patient sub-groups may provide better results (as the selected PSA data closer correspond to those of the case under prediction). Obviously, by applying *w_1_* weighting, a series of customized PSAs can be pre-calculated before prediction.

Multi-parameter prediction is also feasible and could potentially improve the outcome. For instance, the concurrent use of the low-thresholded infarct frequency map, Figure 3d (i.e., 0 for low and 1 for the remaining frequencies) multiplied by a predicted parameter map could reduce outliers by eliminating from prediction the PSA regions with a few cases only.

Prediction of individual mRS scores is feasible and the case selection operation improves it. Although, generally, it is known that it is hard to predict more severe cases [12], our results indicate that this may be feasible, as the PSA-based prediction error for mild and severe outcomes (mRS = 2,…,5) is between 0.5–0.7 (Table 3).

The dichotomical favourable versus unfavourable classification with the PSA is also feasible (Table 4), and the areas under curves could improve with removal of low infarct frequency values.

### Stroke atlas comparison

Our probabilistic atlas differs from the other efforts aiming to develop stroke atlases. A 3D stroke atlas [38] correlates disorders with neuroanatomy by linking a cerebrovascular lesion location with the resulting disorder along with the corresponding signs, symptoms and/or syndromes. A probabilistic atlas [39] created from 22 cases provides a spatial distribution of acute infarcts (it is a special case of the PSA for the image density only and without weighting and case selection). To quantify the impact of infarct location on stroke severity, Menzes et al. [40] constructed brain atlases composed of location-weighted values from 80 ischemic stroke patients. Predefined anatomical regions (but not infarcts) were weighted depending on their size and NIHSS. Note that these existing probabilistic stroke-related atlases use a smaller number of cases than that in our atlas.

### Prediction approach comparison

The existing stroke outcome prediction approaches differ in terms of prognostic models, risk scores, number of independent variables, and predicted scores, among others. There are at least 110 stroke and cardiovascular disease risk scoring methods [26]. The stroke outcome prediction methods range from layperson-oriented models [21] to quick and easy-to-perform scales [14], [18] to regression-based models [7] to stroke risk (point- and web-based) calculators [24] to infarct volume-based prediction [25] and to examinations requiring specialized kits to measure biochemical parameters, such as free triiodothyronine [9] or serum tight-junction proteins for clinically significant hemorrhagic transformation measurement [10]. The majority of prediction models are clinical-based versus layperson-oriented models, which do not require a clinic visit and contain modifiable lifestyle and behavioural parameters [21].

A range of predicted outcomes includes risk of intracranial hemorrhage after thrombolysis [15], [18], [19], [20], poor prognosis and severe disability [6], [9], functional outcome after thrombolysis [7], [17], risk of hemorrhagic transformation [10], short- and long-term mortality [5], [6], [28], long-term outcome [8], hospital disposition [22], ischemic stroke recurrence [23], acute stroke outcome [13], [16], [27], and incidence of ischemic stroke [21]. The number of independent variables varies, e.g., from two variables only (age and NIHSS) [14] to six simple variables (age, living alone, independence in activities of daily living before the stroke, verbal component of the Glasgow Coma Scale, arm power, and ability to walk) [11] to numerous variables (e.g., 12 in mortality prediction [5]).

Some works, such as [41], [42], also use the ROC curves to assess binary classification. Asadi et al. used 107 consecutive acute anterior circulation ischemic stroke patients to evaluate a binary classifier for potential good (mRS≤2) and poor (mRS>2) outcomes and report the area under ROC of 0.6 [41]. Our approach gives a better accuracy, as this area for distinguishing mRS7 outcome of 0–2 versus 3–6 is 0.779 and that for distinguishing 0–1 versus 2–6 is 0.704. For mRS30, the corresponding areas are 0.71 and 0.72. This shows a promising potential of our approach. By applying it to the ACA, MCA, and PCA territories, the ROC area may potentially be improved.

Weimar et al. used a data pool of 9849 patients collected in 23 neurology departments [42]. The prediction concerned complete restitution (BI≥95) versus incomplete restitution or mortality (BI<95). For a 0.437 threshold, the ROC gives correct classification for 80.7% patients. The model is based on conventional logistic regression which does not take location into account. Our method, when assessing favourable (BI≥46) versus unfavourable (BI<46) outcome prediction for BI180, resulted in the area under ROC of 0.85.

### PSA advantages

Our approach conceptually differs from the abovementioned efforts (as illustrated in Figure 1), although it is complementary to and can be combined with them. It is ischemic infarct size and location-driven and combines neurological with neuroradiological approaches by correlating neurological parameters with diagnostic scans in a population. The PSA for a given neurological parameter represents its spatial distribution across the brain, aggregated by the case selective and infarct region weighted accumulation for a population of stroke patients. The prediction is based on obtaining the distribution of this parameter in the normalized infarcted region of the case under prediction. The resulting PSA not only gives insights into the nature of an ischemic lesion distribution (see, e.g., Figure 3d) but also into a parameter distribution (e.g., Figures 3a, b, c) and it enables outcome prediction simultaneously for multiple parameters.

Weighting fine tunes the process of data aggregation by imposing a penalty to reduce the influence of a non-overlapping part of a PSA contour file onto a predicted case. Weighting is also applicable as a selection operation to identify the most relevant cases to build the PSA. Theoretically, the most desirable weighting is *w*_3_ restricted to cases satisfying *w*_3_≈2 (meaning that the selected PSA contour files are very close or same to that of the to-be-predicted case).

As the PSA contains time-specific maps, prediction over time is potentially feasible. The case selection operation in PSA construction enables the inclusion of specific patients allowing the computation of PSAs for patient subgroups. As the PSA is a stereotactic atlas located in the Talairach space, it can be combined with anatomical [31], [43] and blood supply territories [43] atlases. The PSA is a dynamic atlas, easily updatable with new cases.

In this work, a preliminary PSA was calculated and analyzed for ischemic lesions imagined on NCCT; however, the proposed method is general and any parameters may be linked with any imaging data, not only structural but also connectional by the use of diffusion tensor imaging to assess the integrity of white matter pathways and functional imaging to study patterns of cortical activity [12].

Despite a time consuming simulation and analyses performed here for numerous parameters and a huge number of combinations, the actual PSA-based prediction is fast and takes a few seconds only.

### Limitations

This work has two major types of limitations: one due to the method and another one due to the available data. The method requires the lesion of a case, either forming the PSA or to be predicted, to be delineated, meaning that the lesion has to be visible. This may not be feasible in hyperacute stage on NCCT, and these cases cannot be used and predicted. We also assumed no mass displacement and midsagittal plane shift to enable using a fast normalization method due to a huge number of combination analyzed. We employed a low degrees of freedom (DOF) transformation for spatial normalization. This approach is rapid and works with sparse data. The use of a high DOF warping techniques, such as those reviewed in [44], could potentially improve the predictability of the PSA, though increasing the time of PSA calculation, which may be an issue when the number of cases is large. Moreover, these techniques are mostly applicable to magnetic resonance imaging, whereas our statistically-based approach works for any acquisition, including sparse NCCT and does not require scan interpolation.

Though the number of times the CT scans were processed was vast (about 75 millions), the number of actual cases (patients) was still relatively small because of the strict case selection criteria (which reduced the initial dataset almost 4 times) aiming to choose the most relevant and accurate cases to build the preliminary PSA and to perform this proof of concept study. The PSA was computed here for 11 parameters only, whereas 9 parameters were used for prediction (in fact, practically, we employed 2 outcome parameters for prediction and reported the results in Tables 2, 3, 4, as the mRS and BI scores were combined over time; all 11 parameters were illustrated only as maps in Figure 3). The current PSA was created for generally treated patients. To provide prediction of functional outcome after thrombolysis, adequate data shall be collected and suitable probabilistic maps built.

Although the patients were followed-up for one year in terms of outcome, causes of mortality or morbidity other than stroke potentially influencing this outcome were not recorded and taken into account in this study.

The currently constructed PSA was limited to “pure” ischemic infarcts to facilitate the analyses, so cases with leukoaraiosis, old infarcts, hemorrhage, edema and mass effect were not included. By including other pathologies, potentially more specific and clinically useful atlases can be constructed. Cases with mass effect causing an anatomical distortion of the interhemispheric fissure were not included to avoid misregistration errors, as the method used for image normalization is based on an automatic detection of the midsagittal plane. This method is very fast making all 75 million normalizations feasible in a reasonable time.

### Future work

We aim to construct more powerful and specific PSAs, quantify and validate them as well as compare with the existing methods. Such validation will be essential before the PSA can be considered adequate for any clinical use. For this purpose, large cohorts of patients shall be employed.

As anatomical localization is vital [38], [40], the PSA will be combined with our anatomical [31], [43], blood supply territories [43] and stroke [38] atlases. The PSA will be built with a higher sampling rate along the third axis to get isotropic PSA volumes.

The current prediction procedure requires the determination of the contour file of the predicted case, which in this work has been delineated manually. Infarct localization and its volume estimation can be determined automatically [30], which will expedite the procedure.

### Summary

We introduced a novel stroke outcome prediction method based on the “same-parameters-different-patients” model. This method is lesion size and location-driven and uses a Population-based Stroke Atlas (PSA). The PSA links neurological parameters with pathology localized on neuroimages for a population of stroke patients. It aggregates a multiplicity of parameters and presents the distribution of each parameter as a 3D image.

The properties of the PSA were studied for different data aggregation and data selection schemes. We examined eight data aggregation schemes expressed in terms of variants (weights). The prediction outcomes of all eight PSA variants were statistically equivalent. Computationally, the most efficient and simplest was the *w_1_* variant aiming at parameter averaging, and this PSA variant was used to study PSA-based prediction. This variant also allows the PSA to be pre-calculated before prediction, which is not feasible for the other 6 PSA variants. Moreover for the *w_1_* variant, a multiplicity of PSAs can be computed independently for different datasets at various centers and easily merged, which enables building powerful PSAs over the community.

We demonstrated that the data selection process improved outcome accuracy. The PSA constrained by 2 variables reduced the average prediction error by a fraction of 0.796 and the PSA constrained by 3 variables further lowered this error by a fraction of 0.538.

By employing a preliminary version of the PSA, we demonstrated that prediction of individual mRS and BI scores was feasible. Despite a known difficulty in predicting more severe cases, our results indicated that this might be feasible with our method, as the PSA-based prediction error for mild and severe outcomes (mRS = 2,…,5) was between 0.5–0.7.

We also demonstrated the feasibility of the dichotomical classification by means of our method to distinguish favourable (mRS≤2) from unfavourable (mRS>2) outcomes. The highest value of the area under ROC was of 0.779, while that reported recently in PLoS One was of 0.6.
